# 6,7-Difluoro-1,2,3,4-tetra­hydro­quin­oxa­line-5,8-dicarbonitrile

**DOI:** 10.1107/S1600536813003206

**Published:** 2013-02-13

**Authors:** Bao-Hua Qu, Xiao-Chuan Jia, Jing Li, Ming-Yang He

**Affiliations:** a Key Laboratory of Fine Petrochemical Technology, Changzhou University, Changzhou 213164, People’s Republic of China; b Tianjin Entry-Exit Inspection and Quarantine Bureau, Tianjin 300457, People’s Republic of China

## Abstract

In the title compound, C_10_H_6_F_2_N_4_, the C_ar_—N bonds are slightly shortened with respect to a standard aniline C—N bond [1.3580 (16) and 1.3618 (16) *versus* 1.39 Å], thus indicating some π–π conjgation with the electron-acceptor CN groups. The mol­ecule, except for two C atom of the ethyl­ene bridge, is nearly planar, the largest deviation of the other non-H atoms from the mean plane being 0.309 (2) Å. The N—C—C—N torsion angle involving the ethyl­ene bridge is 50.23 (18)°. In the crystal, mol­ecules are connected by pairs of N—H⋯N hydrogen bonds into chains along [21-1].

## Related literature

For general background to the synthesis and use of tetra­fluoro­terephthalonitrile and its derivatives, see: Meazza *et al.* (2007 ▶). For reference structural data on tetra­fluoro­terephthalic acid, see: Orthaber *et al.* (2010 ▶). For standard bond lengths, see: Allen *et al.* (1987 ▶). For hydrogen bonding graph-set descriptors, see: Etter (1990 ▶).
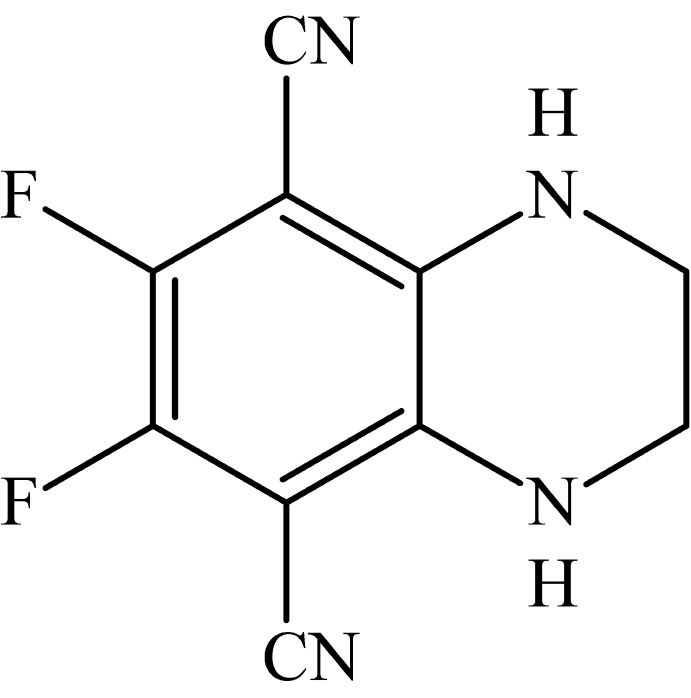


## Experimental

### 

#### Crystal data

C_10_H_6_F_2_N_4_
*M* *_r_* = 220.19
Triclinic, 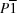

*a* = 5.2173 (9) Å
*b* = 8.7011 (15) Å
*c* = 11.1453 (19) Å
α = 75.545 (2)°
β = 81.854 (2)°
γ = 76.427 (2)°
*V* = 474.40 (14) Å^3^
*Z* = 2
Mo *K*α radiation
μ = 0.13 mm^−1^
*T* = 293 K
0.28 × 0.24 × 0.16 mm

#### Data collection

Bruker APEXII CCD diffractometer
Absorption correction: multi-scan (*SADABS*; Sheldrick, 2003 ▶) *T*_min_ = 0.964, *T*_max_ = 0.980
4155 measured reflections
2141 independent reflections
1816 reflections with *I* > 2σ(*I*)
*R*_int_ = 0.026

#### Refinement

*R*[*F* ^2^ > 2σ(*F* ^2^)] = 0.043
*wR*(*F* ^2^) = 0.140
*S* = 1.06
2141 reflections
145 parameters
H-atom parameters constrained
Δρ_max_ = 0.29 e Å^−3^
Δρ_min_ = −0.23 e Å^−3^

### 

Data collection: *APEX2* (Bruker, 2007 ▶); cell refinement: *SAINT* (Bruker, 2007 ▶); data reduction: *SAINT*; program(s) used to solve structure: *SHELXTL* (Sheldrick, 2008 ▶); program(s) used to refine structure: *SHELXTL*; molecular graphics: *SHELXTL* and *DIAMOND* (Brandenburg, 2005 ▶); software used to prepare material for publication: *SHELXTL*.

## Supplementary Material

Crystal structure: contains datablock(s) I, global. DOI: 10.1107/S1600536813003206/yk2078sup1.cif

Structure factors: contains datablock(s) I. DOI: 10.1107/S1600536813003206/yk2078Isup2.hkl

Supplementary material file. DOI: 10.1107/S1600536813003206/yk2078Isup3.cml

Additional supplementary materials:  crystallographic information; 3D view; checkCIF report

## Figures and Tables

**Table 1:** Hydrogen-bond geometry (Å, °)

*D*—H⋯*A*	*D*—H	H⋯*A*	*D*⋯*A*	*D*—H⋯*A*
N3—H3⋯N1^i^	0.86	2.29	3.075 (2)	152
N4—H4⋯N2^ii^	0.86	2.21	3.0358 (19)	160

Symmetry codes: (i) 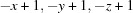; (ii) 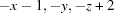.

**Fig. 1.**
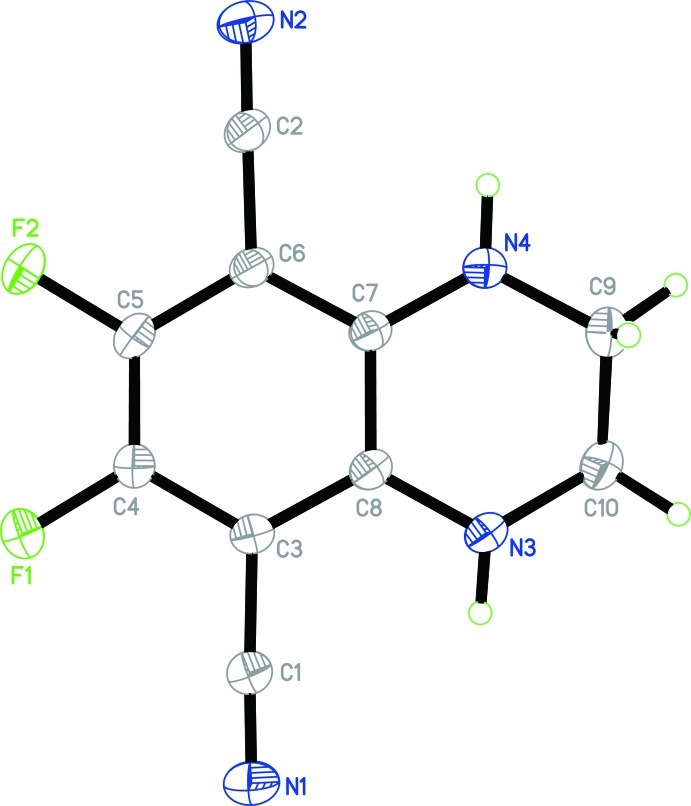
The molecular structure of the title compound showing the atom labelling scheme. Thermal ellipsoids are drawn at the 30% probability level.

**Fig. 2.**
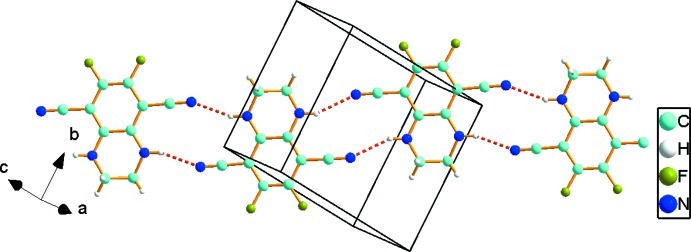
1-D hydrogen-bonding tape of (I) formed by N–H···N interactions. Hydrogen bonds indicated by dashed lines.

## supplementary crystallographic information

### Comment

As important organic intermediates, tetrafluoroterephthalonitrile and its hydrolyzed product tetrafluoroterephthalic acid can be used to prepare pesticide tefluthrin (Meazza *et al.*, 2007; Orthaber *et al.*, 2010). The S_N_Ar reaction of tetrafluoroterephthalonitrile with ethylenediamine under ultrasound irradiation yields 6,7-difluoro-1,2,3,4-tetrahydroquinoxaline-5,8-dicarbonitrile [C_10_H_6_F_2_N_4_, compound (I)] as the main product. While the crystal structure of compound (I) was suspiciously unknown. Herein, we report the crystal structure of (I) for comparison and reference purposes.

Compound (I) crystallizes in triclinic *P*1 space group. A perspective view of the title compound (I) is shown in Fig. 1. The bond lengths and angles are within normal ranges. In the molecule, two nitrile groups are nearly coplanar with the central benzene plane. Within the tetrahydroquinoxaline ring, the torsion angle N3–C10–C9–N4 is -50.23 (18)°. Hydrogen-bonding interactions between the imino groups and cyano groups give rise to cyclic system of two N–H···N bonds between two adjacent molecules with the graph-set motif *R*_2_^2^(12) (Etter, 1990). Due to the chemical symmetry of the molecule itself, such hydrogen-bonding interactions link the molecules to form a one-dimensional (1-D) tape structure (Fig. 2).

### Experimental

Compound (I) was synthesized by the ultrasound reaction of tetrafluoroterephthalonitrile and ethylenediamine at room temperature in the presence of sulfur and assisted by ultrasound irradiation. The title compound was purified through column chromatography with ethyl acetate/petroleum ether as the eluent. Qualified crystalline samples were obtained through slow evaporation from the EtOH solution of (I).

### Refinement

All H atoms were positioned geometrically (C–H = 0.97 Å, N–H = 0.86 Å) and included in the refinement in the riding-model approximation, with *U*_iso_(H) = 1.2*U*_eq_(C,N).

### Crystal data

**Table:**

C_10_H_6_F_2_N_4_	*Z* = 2
*M**_r_* = 220.19	*F*(000) = 224
Triclinic, *P*1	*D*_x_ = 1.541 Mg m^−^^3^
Hall symbol: -P 1	Mo *K*α radiation, λ = 0.71073 Å
*a* = 5.2173 (9) Å	Cell parameters from 2503 reflections
*b* = 8.7011 (15) Å	θ = 2.5–27.6°
*c* = 11.1453 (19) Å	µ = 0.13 mm^−^^1^
α = 75.545 (2)°	*T* = 293 K
β = 81.854 (2)°	Block, colorless
γ = 76.427 (2)°	0.28 × 0.24 × 0.16 mm
*V* = 474.40 (14) Å^3^	

### Data collection

**Table:**

Bruker APEXII CCD diffractometer	2141 independent reflections
Radiation source: fine-focus sealed tube	1816 reflections with *I* > 2σ(*I*)
Graphite monochromator	*R*_int_ = 0.026
φ and ω scans	θ_max_ = 27.6°, θ_min_ = 1.9°
Absorption correction: multi-scan (*SADABS*; Sheldrick, 2003)	*h* = −6→6
*T*_min_ = 0.964, *T*_max_ = 0.980	*k* = −11→11
4155 measured reflections	*l* = −14→13

### Refinement

**Table:**

Refinement on *F*^2^	Primary atom site location: structure-invariant direct methods
Least-squares matrix: full	Secondary atom site location: difference Fourier map
*R*[*F*^2^ > 2σ(*F*^2^)] = 0.043	Hydrogen site location: inferred from neighbouring sites
*wR*(*F*^2^) = 0.140	H-atom parameters constrained
*S* = 1.06	*w* = 1/[σ^2^(*F*_o_^2^) + (0.0825*P*)^2^ + 0.0701*P*] where *P* = (*F*_o_^2^ + 2*F*_c_^2^)/3
2141 reflections	(Δ/σ)_max_ < 0.001
145 parameters	Δρ_max_ = 0.29 e Å^−^^3^
0 restraints	Δρ_min_ = −0.23 e Å^−^^3^

### Special details

**Table:**

Geometry. All e.s.d.'s (except the e.s.d. in the dihedral angle between two l.s. planes) are estimated using the full covariance matrix. The cell e.s.d.'s are taken into account individually in the estimation of e.s.d.'s in distances, angles and torsion angles; correlations between e.s.d.'s in cell parameters are only used when they are defined by crystal symmetry. An approximate (isotropic) treatment of cell e.s.d.'s is used for estimating e.s.d.'s involving l.s. planes.
Refinement. Refinement of *F*^2^ against ALL reflections. The weighted *R*-factor *wR* and goodness of fit *S* are based on *F*^2^, conventional *R*-factors *R* are based on *F*, with *F* set to zero for negative *F*^2^. The threshold expression of *F*^2^ > σ(*F*^2^) is used only for calculating *R*-factors(gt) *etc*. and is not relevant to the choice of reflections for refinement. *R*-factors based on *F*^2^ are statistically about twice as large as those based on *F*, and *R*- factors based on ALL data will be even larger.

### Fractional atomic coordinates and isotropic or equivalent isotropic displacement parameters (Å^2^)

**Table:**

	*x*	*y*	*z*	*U*_iso_*/*U*_eq_	
C1	0.5584 (3)	0.24423 (15)	0.57193 (12)	0.0414 (3)	
C2	−0.1573 (3)	−0.09578 (15)	0.89446 (12)	0.0403 (3)	
C3	0.3758 (2)	0.16100 (14)	0.65684 (11)	0.0359 (3)	
C4	0.4178 (2)	−0.00938 (14)	0.67660 (11)	0.0382 (3)	
C5	0.2446 (3)	−0.09029 (14)	0.75315 (12)	0.0389 (3)	
C6	0.0223 (2)	−0.00551 (14)	0.81451 (11)	0.0359 (3)	
C7	−0.0247 (2)	0.16328 (14)	0.79859 (11)	0.0348 (3)	
C8	0.1573 (2)	0.24965 (14)	0.71567 (11)	0.0355 (3)	
C9	−0.2521 (3)	0.41626 (17)	0.85819 (15)	0.0548 (4)	
H9A	−0.1510	0.4206	0.9235	0.066*	
H9B	−0.4347	0.4681	0.8760	0.066*	
C10	−0.1447 (3)	0.50503 (16)	0.73570 (16)	0.0539 (4)	
H10A	−0.2651	0.5195	0.6731	0.065*	
H10B	−0.1288	0.6115	0.7421	0.065*	
F1	0.63601 (16)	−0.08862 (10)	0.61981 (8)	0.0525 (3)	
F2	0.28294 (18)	−0.25305 (9)	0.77522 (9)	0.0548 (3)	
N1	0.6992 (3)	0.31503 (17)	0.50478 (13)	0.0574 (4)	
N2	−0.3029 (3)	−0.16595 (16)	0.95803 (12)	0.0560 (3)	
N3	0.1134 (2)	0.41386 (13)	0.69902 (12)	0.0481 (3)	
H3	0.2066	0.4607	0.6368	0.058*	
N4	−0.2375 (2)	0.24862 (13)	0.85611 (11)	0.0463 (3)	
H4	−0.3500	0.2005	0.9065	0.056*	

### Atomic displacement parameters (Å^2^)

**Table:**

	*U*^11^	*U*^22^	*U*^33^	*U*^12^	*U*^13^	*U*^23^
C1	0.0426 (7)	0.0361 (6)	0.0434 (7)	−0.0099 (5)	0.0025 (5)	−0.0070 (5)
C2	0.0442 (7)	0.0324 (6)	0.0425 (7)	−0.0105 (5)	−0.0031 (5)	−0.0029 (5)
C3	0.0368 (6)	0.0330 (6)	0.0366 (6)	−0.0104 (5)	0.0008 (5)	−0.0046 (5)
C4	0.0385 (6)	0.0328 (6)	0.0413 (6)	−0.0054 (5)	0.0013 (5)	−0.0093 (5)
C5	0.0450 (7)	0.0263 (5)	0.0438 (7)	−0.0075 (5)	−0.0038 (5)	−0.0050 (5)
C6	0.0384 (6)	0.0307 (6)	0.0372 (6)	−0.0106 (5)	−0.0021 (5)	−0.0029 (5)
C7	0.0348 (6)	0.0307 (6)	0.0367 (6)	−0.0080 (4)	−0.0009 (5)	−0.0038 (4)
C8	0.0374 (6)	0.0290 (6)	0.0389 (6)	−0.0091 (4)	−0.0014 (5)	−0.0043 (4)
C9	0.0552 (8)	0.0362 (7)	0.0657 (9)	−0.0042 (6)	0.0124 (7)	−0.0132 (6)
C10	0.0528 (8)	0.0307 (6)	0.0715 (10)	−0.0053 (5)	0.0069 (7)	−0.0091 (6)
F1	0.0486 (5)	0.0416 (4)	0.0609 (5)	−0.0030 (3)	0.0120 (4)	−0.0149 (4)
F2	0.0620 (5)	0.0262 (4)	0.0709 (6)	−0.0087 (3)	0.0058 (4)	−0.0084 (4)
N1	0.0592 (8)	0.0508 (7)	0.0578 (7)	−0.0212 (6)	0.0136 (6)	−0.0053 (6)
N2	0.0555 (7)	0.0487 (7)	0.0587 (7)	−0.0205 (6)	0.0065 (6)	0.0002 (6)
N3	0.0470 (6)	0.0279 (5)	0.0627 (7)	−0.0106 (4)	0.0132 (5)	−0.0051 (5)
N4	0.0420 (6)	0.0344 (5)	0.0564 (7)	−0.0093 (4)	0.0126 (5)	−0.0075 (5)

### Geometric parameters (Å, º)

**Table:**

C1—N1	1.1425 (17)	C7—C8	1.4354 (16)
C1—C3	1.4330 (17)	C8—N3	1.3618 (16)
C2—N2	1.1408 (17)	C9—N4	1.4485 (18)
C2—C6	1.4310 (17)	C9—C10	1.495 (2)
C3—C8	1.3973 (17)	C9—H9A	0.9700
C3—C4	1.4116 (17)	C9—H9B	0.9700
C4—F1	1.3481 (14)	C10—N3	1.4537 (18)
C4—C5	1.3492 (18)	C10—H10A	0.9700
C5—F2	1.3465 (14)	C10—H10B	0.9700
C5—C6	1.4081 (18)	N3—H3	0.8600
C6—C7	1.4007 (16)	N4—H4	0.8599
C7—N4	1.3580 (16)		
			
N1—C1—C3	177.89 (14)	C3—C8—C7	118.45 (11)
N2—C2—C6	179.11 (14)	N4—C9—C10	110.40 (12)
C8—C3—C4	121.41 (11)	N4—C9—H9A	109.6
C8—C3—C1	119.68 (11)	C10—C9—H9A	109.6
C4—C3—C1	118.89 (11)	N4—C9—H9B	109.6
F1—C4—C5	121.20 (11)	C10—C9—H9B	109.6
F1—C4—C3	118.67 (11)	H9A—C9—H9B	108.1
C5—C4—C3	120.12 (11)	N3—C10—C9	109.85 (12)
F2—C5—C4	121.21 (11)	N3—C10—H10A	109.7
F2—C5—C6	118.54 (11)	C9—C10—H10A	109.7
C4—C5—C6	120.23 (11)	N3—C10—H10B	109.7
C7—C6—C5	121.40 (11)	C9—C10—H10B	109.7
C7—C6—C2	120.11 (11)	H10A—C10—H10B	108.2
C5—C6—C2	118.48 (11)	C8—N3—C10	120.46 (11)
N4—C7—C6	122.84 (11)	C8—N3—H3	114.4
N4—C7—C8	118.78 (11)	C10—N3—H3	118.1
C6—C7—C8	118.36 (11)	C7—N4—C9	121.10 (11)
N3—C8—C3	122.59 (11)	C7—N4—H4	121.1
N3—C8—C7	118.94 (11)	C9—N4—H4	116.3
			
C8—C3—C4—F1	−178.24 (11)	C4—C3—C8—N3	179.46 (12)
C1—C3—C4—F1	3.43 (18)	C1—C3—C8—N3	−2.24 (19)
C1—C3—C4—C5	−177.99 (11)	C4—C3—C8—C7	0.62 (19)
F1—C4—C5—F2	−0.2 (2)	C1—C3—C8—C7	178.93 (10)
C3—C4—C5—F2	−178.78 (11)	N4—C7—C8—N3	1.12 (19)
F1—C4—C5—C6	178.02 (11)	C6—C7—C8—N3	179.76 (11)
C3—C4—C5—C6	−0.5 (2)	N4—C7—C8—C3	180.00 (11)
F2—C5—C6—C7	178.04 (11)	C6—C7—C8—C3	−1.37 (18)
C4—C5—C6—C7	−0.3 (2)	N4—C9—C10—N3	−50.23 (18)
F2—C5—C6—C2	−1.96 (18)	C3—C8—N3—C10	164.48 (13)
C4—C5—C6—C2	179.72 (11)	C7—C8—N3—C10	−16.7 (2)
C5—C6—C7—N4	179.80 (11)	C9—C10—N3—C8	41.7 (2)
C2—C6—C7—N4	−0.20 (19)	C6—C7—N4—C9	167.19 (13)
C5—C6—C7—C8	1.22 (19)	C8—C7—N4—C9	−14.2 (2)
C2—C6—C7—C8	−178.78 (10)	C10—C9—N4—C7	39.5 (2)

### Hydrogen-bond geometry (Å, º)

**Table:**

*D*—H···*A*	*D*—H	H···*A*	*D*···*A*	*D*—H···*A*
N3—H3···N1^i^	0.86	2.29	3.075 (2)	152
N4—H4···N2^ii^	0.86	2.21	3.0358 (19)	160

Symmetry codes: (i) −*x*+1, −*y*+1, −*z*+1; (ii) −*x*−1, −*y*, −*z*+2.
